# Soil Fungal Community Diversity, Co-Occurrence Networks, and Assembly Processes under Diverse Forest Ecosystems

**DOI:** 10.3390/microorganisms12091915

**Published:** 2024-09-20

**Authors:** Bing Yang, Zhisong Yang, Ke He, Wenjia Zhou, Wanju Feng

**Affiliations:** Sichuan Academy of Giant Panda, Chengdu 610081, China; yangzhisong@126.com (Z.Y.); heke0611@126.com (K.H.); wenjia851209@163.com (W.Z.); 19828320401@163.com (W.F.)

**Keywords:** fungal functional guilds, β diversity, co-occurrence network, deterministic and stochastic processes

## Abstract

Fungal communities are critical players in the biogeochemical soil processes of forest ecosystems. However, the factors driving their diversity and community assembly are still unclear. In the present study, five typical vegetation types of soil fungal communities in Liziping Nature Reserve, China, were investigated using fungal ITS sequences. The results show that the topsoil fungal community is mainly dominated by the phyla Ascomycota, Basidiomycota, and Mortierellomycota. Although there was no significant difference in α diversity (Shannon, Simpson, and Pielou evenness indices) among different forest types, there was a significant difference in β diversity (community composition). This study found that soil pH, soil organic carbon, total nitrogen (TN), total phosphorus (TP), and the total nitrogen/total phosphorus (N/P) ratio are the main environmental factors that affect soil fungal communities. Each forest type has a specific co-occurrence network, indicating that these community structures have significant specificities and complexities. Deciduous evergreen broad-leaved forests as well as deciduous broad-leaved and evergreen broad-leaved mixed forests showed high modularity and average path lengths, indicating their highly modular nature without distinct small-scale characteristics. Furthermore, our findings indicate that the structures of topsoil fungal communities are mainly shaped by stochastic processes, with the diffusion limitation mechanism playing a particularly significant role.

## 1. Introduction

Fungi play essential roles in biogeochemical cycling, plant nutrition, primary productivity, carbon mineralization, sequestration, and disease management [1,2,3]. For example, fungal composition can indicate biotic conditions and can be used to predict overall forest carbon storage [4]. They also serve as indicators of soil quality [5]. In natural ecosystems, soil fungi and plants engage in close interactions [6]. Dominant plant species influence the composition of soil fungal communities [7,8], while the microhabitats are formed by different vegetation types appropriate for specific microbial colonization patterns [9,10]. Conversely, soil fungal communities impact plant community composition and diversity [6,11,12,13]. These reciprocal relationships highlight the complex dynamics within forest ecosystems. Recent studies have shown that specific soil fungi can enhance plant resilience to environmental stressors, such as drought and pathogens [14,15,16,17,18]. Additionally, such as those involving carbon, nitrogen, and phosphorus, may be linked to changes in fungal community composition, abundance, and diversity [19]. Understanding these complex interactions is critical for developing forest conservation and management strategies aimed at preserving ecosystem balance and biodiversity [7].

At a functional guild level, soil fungi can be mainly classified as symbiotrophic, saprotrophic, and pathotrophic [20]. These functional guilds carry out distinct ecological functions based on their specific trophic strategies [21]. For instance, symbiotrophic fungi (including arbuscular mycorrhizal fungi (AMF) and ectomycorrhizal fungi (EMF)) enhance plant nutrient absorption and increase resistance to stressors. Saprotrophic fungi decompose litter, providing a source of carbon and energy for growth [22,23]. Pathogenic fungi can increase host susceptibility to fungal infections and insect infestations [24,25,26], impeding plant growth and reproduction. Studies have shown that distinct fungal guilds interact synergistically to facilitate multiple ecosystem processes [27,28,29], such as stabilizing plant diversity and community composition and providing forest ecosystem functioning [30]. For example, interactions between saprotrophs and AMF enhance plant fitness in conditions of low soil nutrient availability by promoting litter decomposition [6,27]. However, due to differences in dependence on aboveground plants and sensitivity to changes in environmental factors [2], distinct functional guilds respond differently to changes in the same environmental conditions. Consequently, shifts in fungal functional guilds can significantly impact ecosystem functioning, such as forest stand productivity [31] and soil nutrient cycling [32].

Recent advances in high-throughput sequencing technologies have revolutionized our ability to characterize soil fungal communities with unprecedented resolution and accuracy [1]. These tools can help decipher the roles of rare or unculturable species, shedding light on their contributions to ecosystem functions and their responses to environmental changes. Additionally, ecological network analysis provides an efficient approach to investigate potential interactions between microbial taxa [33]. Ultimately, integrating these molecular approaches with ecological theory and long-term empirical studies will allow us to develop a predictive framework for fungal community assembly. Such a framework will be invaluable in guiding conservation efforts and informing sustainable forest management practices that aim to preserve the integrity of soil ecosystems and the services they provide. It is broadly recognized that community assembly is influenced by both deterministic and stochastic processes [34,35]. Previous studies showed that soil fungal communities, with the exception of mycorrhizal fungi, were primarily controlled by drift [34,36,37,38]. Previous studies have also demonstrated that the relative influence of deterministic and stochastic processes on fungal community composition changes according to geography [39], environmental factors, taxonomy groups [40], and stressors [41].

Forests are among the most diverse ecosystems on Earth, harboring a rich microbial diversity that is crucial for ecosystem functioning and services [1]. Forest ecosystems can be broadly categorized into several types, each with distinct characteristics and management practices. Broadleaf forests, dominated by deciduous trees, tend to have rich organic layers and diverse microbial communities [42]. Coniferous forests, composed mainly of evergreen trees, often have acidic soils with unique fungal assemblages adapted to these conditions [43]. Conifer forest soil contain high cellulose and low lignin contents [30], which are conducive to the differentiation of saprophytic fungi and ectomycorrhizal communities [26]. Mixed forests, containing both broadleaf and coniferous species, display diversified soil resource pools, high soil heterogeneity, reduced competition pressure within the microbial community, and high diversity [9]. Secondary forests, which regrow after significant disturbances, and plantation forests, established through human intervention, present unique opportunities to study successional changes and human impacts on soil microbial communities [44,45].

Soil fungal communities are influenced by numerous factors, including altitude [46], soil properties [47], vegetation/forest type [7,21,48,49], and soil type [50]. Soil properties, particularly soil pH [1,51], and soil nutrients, such as phosphorus (P) content [32,50,52], regulate soil fungal communities. Forest type, characterized by differences in dominate tree species, plant diversity and associated environmental conditions, may directly affect fungal communities or indirectly affect fungal communities through changing microclimate and soil properties [21,50]. However, the interaction between forest type and soil fungal community is very complex and variable. Most available studies indicate that forest type influences soil fungal diversity [21,48,49]. However, some studies have reported that changes in vegetation types do not affect soil fungal diversity [53]. Generally, the drivers of soil fungal communities vary with functional group [21,25], and even the drivers of the same functional groups of soil fungal communities differ among various habitats [54].

In the present study, the community composition, functional guilds, diversity, co-occurrence network, and assembly processes were determined using the Illumina MiSeq sequencing and bioinformatics mining platform. The following questions were addressed: (1) Is vegetation type the key factor that affects soil fungal community structure, functional guilds, and diversity? (2) Are there differences in soil fungal co-occurrence networks under different vegetation types? (3) What is the soil fungal community assembly process in this region? By integrating high-throughput sequencing, network analysis, and community assembly models, this study provides insights into the patterns and drivers of soil fungal diversity in different forest ecosystems. It was hypothesized that soil fungal and functional group diversity and community composition, as well as co-occurrence networks, were distinct across vegetation types due to vegetation and soil physicochemical properties. Additionally, it was assumed that the soil fungal community assembly process was predominated by deterministic process. The findings of this study are expected to enhance our understanding of soil fungal ecology and inform forest management practices to promote ecosystem resilience and biodiversity.

## 2. Materials and Methods

### 2.1. Study Site

The study site is located at the Shimian County, Yanan City (102°10′33″–102°29′07″ E, 28°51′02″–29°08′42″ N), Sichuan Province, China. The mean annual temperature and mean annual precipitation are 11.7–14.4 °C and 800–1250 mm, respectively. The soil type of this area is braunerde, or brown forest soil [55]. The typical vegetation types include evergreen broadleaf forests, mixed coniferous and broadleaf forests, deciduous broadleaf and evergreen broadleaf mixed forests (secondary forests), coniferous forests, and plantation forests. The evergreen broadleaf forest is found at altitudes below 2400 m. The dominant tree species in this forest type are *Lindera limprichtii*, *Phoebe chinensis*, *Cyclobalanopsis oxyodon*, *Quercus engleriana*, and *Lithocarpus cleistocarpus*, with a few deciduous tree species present, such as *Euptelea pleiospermum* and *Corylopsis willmottiae*, which are distributed sporadically. The secondary forest is primarily located at altitudes of 2000–2500 m above sea level, featuring dominant evergreen tree species such as *Cyclobalamopsis gracili*, *Quercus oxyodon*, *Quercus aliena*, and *Lithocarpus lieistocarpus*, while the dominant deciduous tree species include *Pterocarya insignis*, *Juglans cathayensis*, and *Populus lasiocarpa*. The mixed coniferous and broadleaf forests are mainly found at altitudes ranging from 2500 m to 2700 m above sea level, originally composed of *Tsuga chinensis*, *Picea brachytyla, Acer tetramerum*, *Acer laxiflorum, Acer caudatum*, *Acer maximowiczii*, and *Betula utilis*. The staple bamboo species of giant pandas are *Fargesia ferax* and *Bashania spanostachya*. The coniferous forest is predominantly found at 3100–3200 m above sea level, with dominant tree species such as *Tsuga chinensis, Picea asperata*, and *Abies fabri*. The understory bamboo species that serves as staple food for giant pandas is *Arundinaria spanostachya.* The plantation forest mainly distributed at 2000–2300 m above sea level, with dominant tree species including *Cryptomeria japonica* and *Larix kaempferi*. There are few understory plants in these forests, and no staple bamboo species for giant pandas are present.

### 2.2. Soil Sampling

Soil sampling was conducted in October 2020 to minimize seasonal variations. Briefly, soil samples were collected from each forest site using a systematic sampling approach. At each site, 10 soil cores were collected at random locations, with each core representing a unique sampling point. Cores were collected at depths of 0–20 cm using a soil corer and combined to form a composite sample. Soil samples were transported in an ice box to the laboratory. After the removal of gravel and visible plant residues, soil samples were mixed thoroughly, passed through a 2 mm sieve, and divided into three portions. One portion was air-dried and ground for the analyses of soil organic carbon (SOC), total nitrogen (TN), total phosphorus (TP), pH, and available phosphorus (AP). The second part of the fresh soil was the soil water content. The measurements of soil physicochemical properties and part of the relevant results have been described previously [55]. The third portion of fresh soil was stored at −80 °C for soil microbial community analyses.

### 2.3. Soil Physicochemical Properties Determination

Soil physicochemical properties were determined with standard protocols. Briefly, soil pH and electrical conductivity (EC) were determined in a soil suspension at a soil/water ratio of 1:2.5 (volume/weight, *v*/*w*), using a digital pH meter (Mettler-Toledo FE28, Mettler Toledo International Inc., Shanghai, China) and conductivity meter (DDS307, Shanghai, China), respectively. The SMC was determined after drying the sieved soil at 105 °C for 48 h in an oven. SOC and TN concentrations were measured using a CHN elemental analyzer (2400II CHN elemental analyzer, PerkinElmer, Boston, MA, USA). Available phosphorus (AP) was analyzed using the molybdenum-antimony colorimetry method following extraction with 0.05 M HCL-0.025 M 1/2 H_2_SO_4_.

### 2.4. Illumina MiSeq Sequencing and Bioinformatics Processing

Fungal DNA was extracted from 0.5 g soil using the OMEGA Soil DNA Kit (M5635-02) (Omega Bio-Tek, Norcross, GA, USA), following the manufacturer’s instructions, and stored at −20 °C prior to further analysis. The quantity and quality of extracted DNA were measured using a NanoDrop NC2000 spectrophotometer (Thermo Fisher Scientific, Waltham, MA, USA) and agarose gel electrophoresis, respectively. DNA was sequenced using an Illumina MiSeq sequencing system (Illumina, San Diego, CA, USA; 2 × 300 base pairs (bp)). The fungal DNA ITS regions were amplified using the commonly used primer pairs ITS1F (5′-GGAAGTAAAAGTCGTAACAAGG-3′) and ITS2 (5′-GCTGCGTTCTTCATCGATGC-3′) [5,56,57]. PCR was performed as follows: initial heating to 98 °C for 5 min, followed by 28 thermal cycles (30 s at 98 °C, 45 s at 55 °C, and 45s extension at 72 °C), and concluded by a 5 min final auto-extension at 72 °C. PCR products were purified using the Axygen DNA Gel Extraction Kit (Axygen Biosciences, Union City, CA, USA) and amplicons were pooled in equal amounts. The PCR mixtures were prepared using the following components: Q5 high-fidelity DNA polymerase, 0.25 μL; 5× Reaction Buffer, 5 μL; 5× High GC Buffer, 5 μL; dNTP(10 mM), 2 μL; DNA templates, 2 μL; forward primer (10μM), 1 μL; reverse primer (10 μM), 1 μL; and ddH2O, 8.75 μL. Paired-end sequencing (2 × 300 bp) was performed using standard protocols by Shanghai Personalbio Technology Co., Ltd. (Shanghai, China), on the Illumina MiSeq sequencing platform (Illumina, San Diego, CA, USA).

### 2.5. Bioinformatics Analysis

Raw sequences were processed using the QIIME 2 pipeline [58] to remove low-quality reads that lacked a valid primer/barcode sequence, contained ambiguous bases, or had an average quality score. Quality filtering, denoising, chimera removal, and amplicon sequence variant (ASV) picking were performed using the DADA2 plugin [59]. The taxonomic assignment of ASVs was conducted using the UNITE database [60] and the naïve Bayes classifier was implemented in QIIME2. The fungal traits dataset (https://github.com/traitecoevo/fungaltraits accessed on 14 June 2024) [61] was used to assign ecological information (primary lifestyle) to ASVs: ECM, ericoid mycorrhiza, arbuscular mycorrhizal, animal endosymbiont, animal parasite, mycoparasite, lichen parasite, litter saprotroph, nectar/tap saprotroph, soil saprotroph, unspecified saprotroph, wood saprotroph, dung saprotroph, plant pathogen, or root endophyte.

### 2.6. Statistical Analyses

A diversity indices, including observed richness, Shannon, Simpson, and Pielou’s evenness indices were calculated to assess fungal community α diversity. Differences in α diversity among forest types were tested using one-way ANOVAs or non-parametric tests, depending on the normality and homogeneity of the variance. If a significant effect was detected, the differences were further assessed using the Tukey HSD test or pairwise *t*-test with the “Benjamini–Hochberg” method to adjust the *p*-value.

For the whole fungal community composition, the non-metric multidimensional scaling (NMDS) and principal coordinate analysis (PCoA) and based on Bray–Curtis distance matrices was used to depict the variation of fungal diversities, followed by a permutational multivariate analysis of variance (PERMANOVA) to test the significance of forest types in microbial community composition. The “vegan” version 2.6-8 package in R version 4.4.1 was used to perform NMDS and PCoA. Venn analysis was also performed to distinguish the number of unique and shared ASVs across five forest types.

To avoid the proposed errors [62], the co-occurrence networks of soil fungal community were constructed using the Sparse Correlations for Compositional data (SparCC) algorithm [63] in the “SpiecEasi” version 1.1.2 package in R [64]. Only the significant correlations between taxa of the top 50 fungal ASVs, both negative and positive (SparCC, weight ≥ 0.6 or ≤−0.6) were represented as edges. Network properties, including connectivity, modularity, and average path length, were calculated using the igraph version 2.0.3 package in R [65]. Additionally, two parameters, within-module degree (Zi) and among-module connectivity (Pi), were further used to characterize the topological roles of different ASVs [66]. The nodes in each network were categorized into (i) network hubs (Zi > 2.5 and Pi > 0.62, highly connected nodes within and among modules), (ii) module hubs (Zi > 2.5, highly connected nodes within a module), (iii) connectors (Pi > 0.62, highly connected nodes among modules), and (iv) peripherals (Zi < 2.5 and Pi < 0.62, nodes with few links or unconnected with other nodes). Keystone species are defined as nodes that act as connectors or hubs in a network.

The relative contributions of deterministic and stochastic processes to fungal community assembly were assessed using the null modeling approach [34]. Briefly, the observed phylogenetic turnover was compared with null models generated by randomizing community compositions while preserving species richness and site occupancy. Deviations of observed turnover from null expectations indicated the relative importance of deterministic and stochastic processes. The null models were run using the algorithms implemented in the iCAMP version 1.5.12 package [35].

## 3. Results

### 3.1. Soil Fungal Community Composition

**Figure 1:**
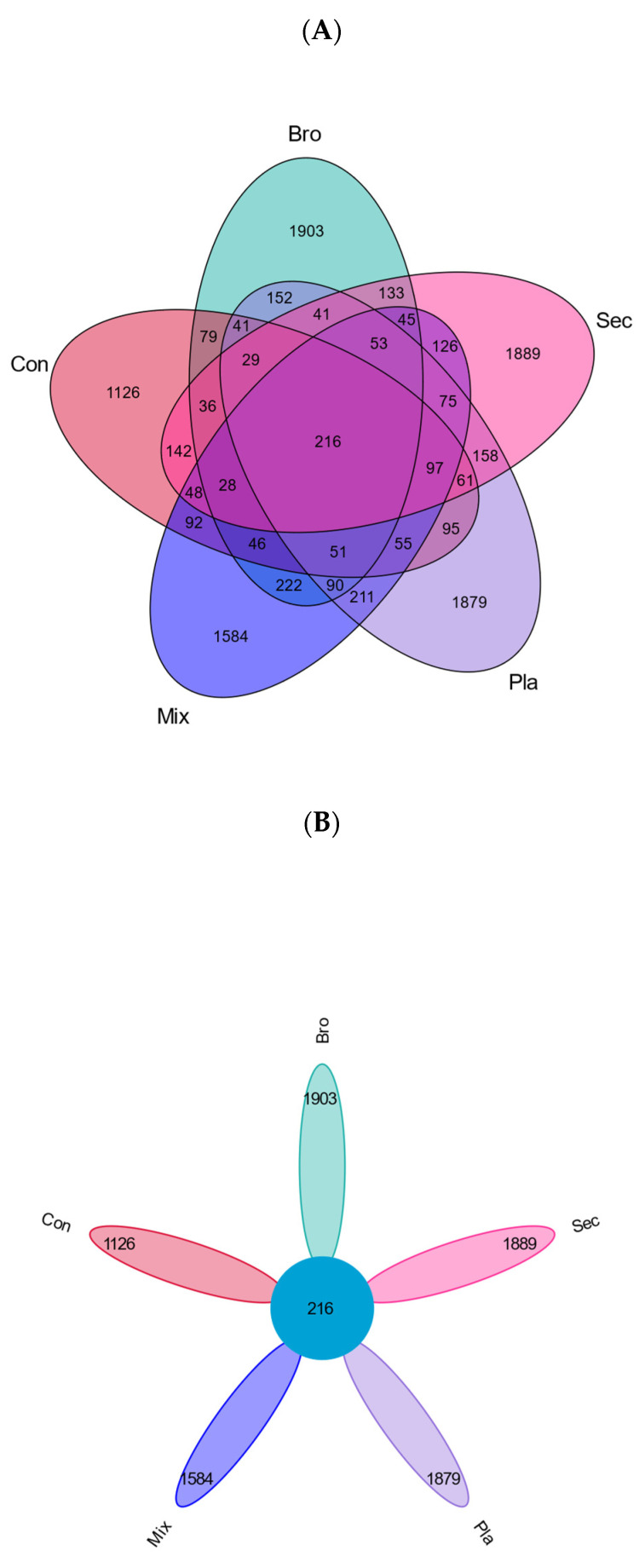
Venn plot (**A**) and flower plot (**B**) of shared and unique fungal ASVs of five examined vegetation types. Bro, evergreen broadleaved forest; Con, coniferous forest; Mix, natural deciduous broadleaved and evergreen broadleaved mixed forest; Sec, secondary forest; Pla, plantation forest.

According to the Venn plot (Figure 1A) and flower plot (Figure 1B), the shared soil fungal ASVs at the topsoil layer among the five examined forest types amounted to 216 (Figure 1A,B). In contrast, the unique soil fungal ASVs for each forest type were as follows: evergreen broad-leaved forest (1903 ASVs), coniferous forest (1126 ASVs), deciduous broad-leaved and evergreen broad-leaved mixed forest (1584 ASVs), secondary forest (1889 ASVs), and plantation forest (1879 ASVs) (Figure 1B).

**Figure 2:**
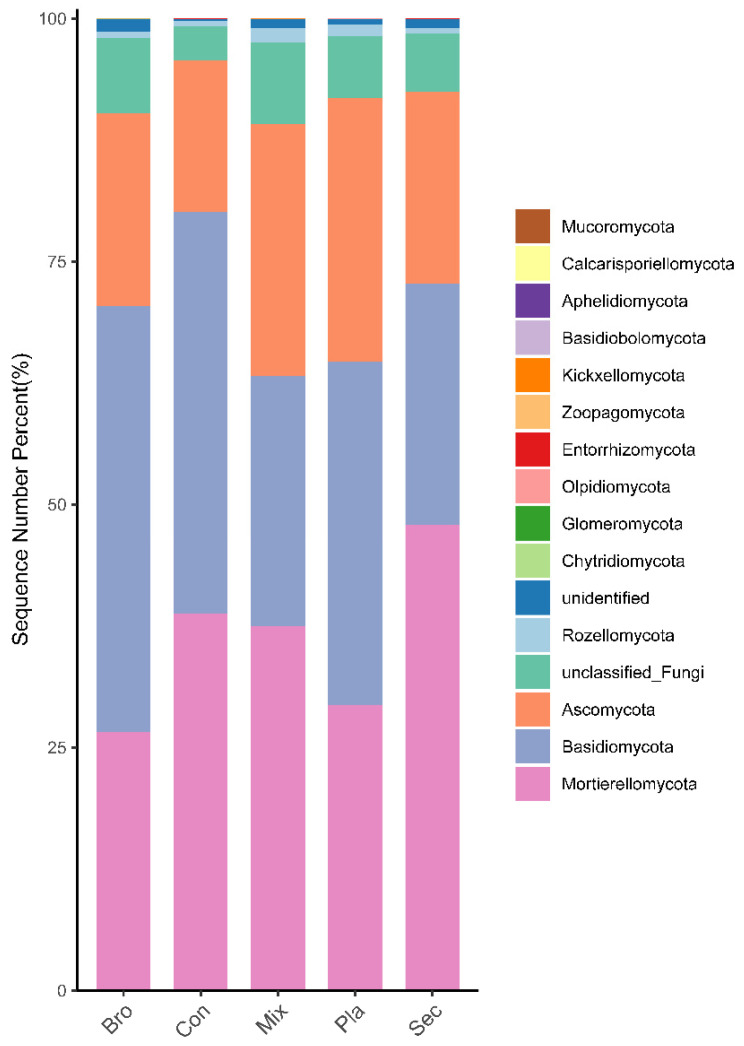
Relative abundance of dominant fungal phyla across five forest types. ‘Others’ include phyla, with <1% average relative abundance. The data represent the mean of nine replicate samples. Bro, evergreen broadleaved forest; Con, coniferous forest; Mix, natural deciduous broadleaved and evergreen broadleaved mixed forest; Sec, secondary forest; Pla, plantation forest.

At the phylum level, the soil fungal communities in the typical forests of the Liziping Natural Reserve were predominantly composed of Ascomycota, Basidiomycota, and Mortierellomycota. However, the composition of these communities varied significantly across the different forest types studied (Figure 2). In the evergreen broad-leaved forests, Basidiomycota was the predominant phylum, making up 43.84% of the total fungal sequences, followed by Mortierellomycota (26.55%) and Ascomycota (19.86%). The coniferous forests exhibited a more balanced distribution among the phyla, with Basidiomycota and Mortierellomycota accounting for 41.32% and 38.77% of the sequences, respectively, and Ascomycota representing 15.59% of the sequences. In the deciduous broad-leaved and evergreen broad-leaved mixed forest, Mortierellomycota, Ascomycota, and Basidiomycota were the predominant phyla, constituting 37.49%, 25.89%, and 25.77% of the total fungal sequences, respectively. The secondary forest was dominated by Mortierellomycota, Basidiomycota, and Ascomycota, making up 47.87%, 24.81%, and 19.83% of the total fungal sequences, respectively. Lastly, in the plantation forest, Basidiomycota, Mortierellomycota, and Ascomycota were the leading phyla, comprising 35.34%, 29.39%, and 27.14% of the total fungal sequences, respectively. Correlation analysis showed that the relative abundance of phylum, guilds, trophic modes, and soil physicochemical properties (Figure S1). The relative abundance of Glomeromycota phylum was significantly positively correlated to soil pH (Figure S1A).

In the trophic group, no significant differences in relative abundance of pathotroph fungi (Figure S2A) and symbiotroph fungi (Figure S2C) were observed across the examined forest types, whereas remarkable differences in saprotroph fungi (Figure S2B) and unassigned fungi (Figure S2D) were observed across the examined forest types. Specifically, the relative abundance of soil saprotroph fungi in secondary forests was significantly higher than that in evergreen broadleaved forests. Additionally, the relative abundance of unassigned soil fungi in plantation forests was significantly higher than in that of secondary forests as well as in that of coniferous forests (Figure S2D). The relative abundance of AMF abundance was significantly positively correlated to soil pH. The relative abundances of Ericoid mycorrhizal fungi, plant pathogen fungi and pathotroph fungi were positively correlated to soil C/P. The relative abundance of plant saprotroph fungi was positively correlated to soil N/P (Figure S1B).

### 3.2. Soil Fungal Community Diversity

#### 3.2.1. Alpha Diversity

**Figure 3:**
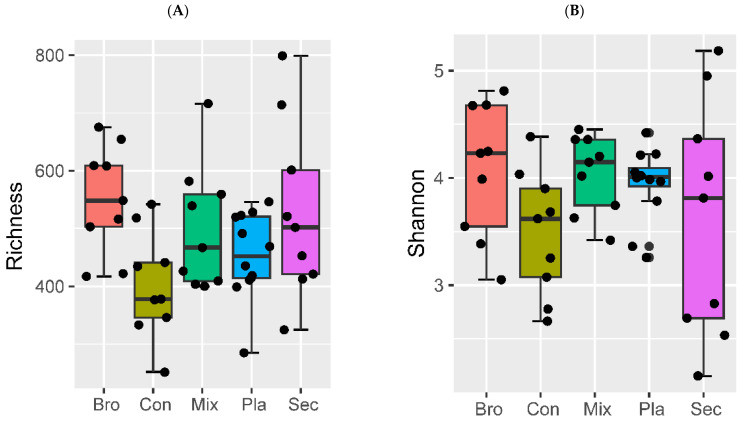

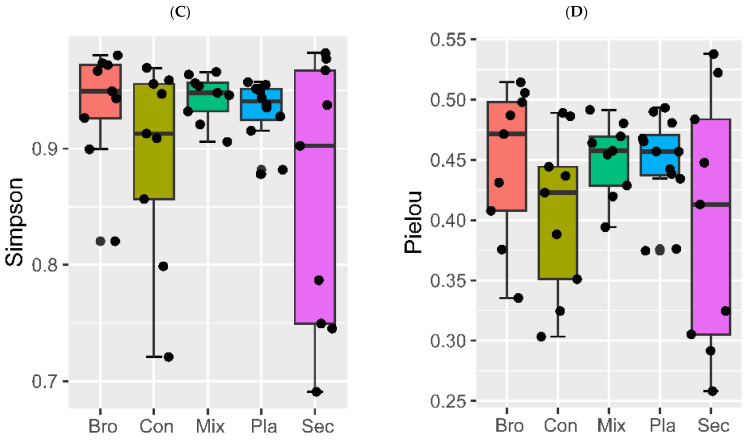
Observed species (**A**), Shannon’s (**B**), Simpson’s (**C**), and Pielou’s evenness (**D**) indices of the α diversity of soil fungal community. The black line indicates the estimated mean effect and the blue bars indicate the 0.95 confidence interval of the mean, while the black bar and dots represent the boxplot and corresponding outliers of the partial residuals. Boxplots denote median value (black line, with hinges (black) representing the 25th and 75th percentiles and lines extending to the 1.5× interquartile range. Bro, evergreen broadleaved forest; Con, coniferous forest; Mix, natural deciduous broadleaved and evergreen broadleaved mixed forest; Sec, secondary forest; Pla, plantation forest.

A diversity metrics, with the exception of the observed species, the Shannon, Simpson, and Pielou evenness indices revealed no significant differences among the forest types (Figure 3A–D). Specifically, the observed fungal species in evergreen broad-leaved forest soil was significantly higher than that in coniferous forest soil.

#### 3.2.2. Beta Diversity

**Figure 4:**
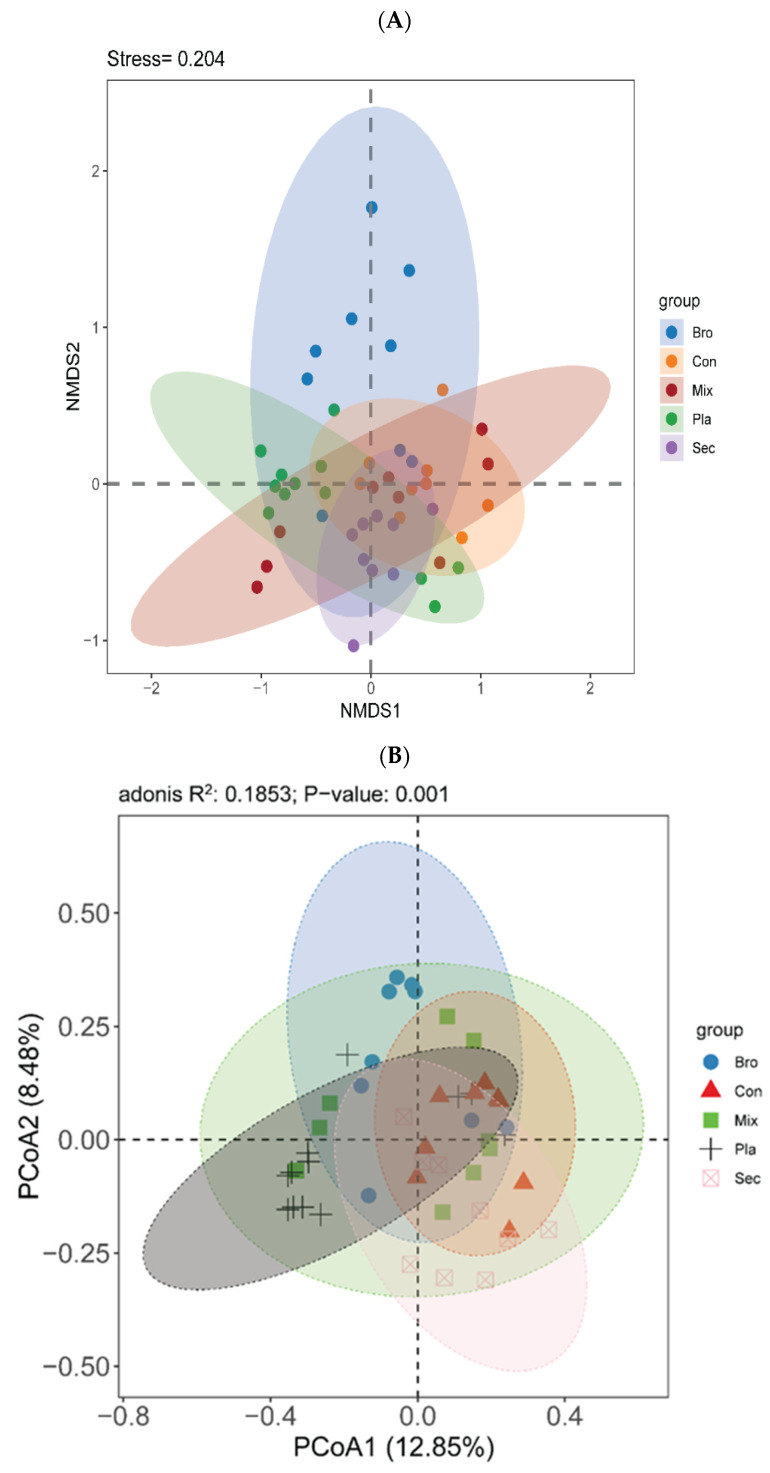
Nonmetric multidimensional scaling analysis (NMDS) of the fungal β diversity based on the Bray–Curtis distance of the ASV matrix (**A**), and different colored dots represent different forest types. The stress value represents a measure of the error between the original distance and the low-dimensional spatial distance obtained using NMDS; soil fungal community compositions in different forest types were revealed by the principal co-ordinates analysis (PCoA) of the fungal β-diversity based on the Bray–Curtis distance of the ASV matrix (**B**). Mantel tests were used to test the significance of correlations between fungal community structure and soil physicochemical properties. Bro, evergreen broadleaved forest; Con, coniferous forest; Mix, natural deciduous broadleaved and evergreen broadleaved mixed forest; Sec, secondary forest; Pla, plantation forest.

The NMDS ordination indicated that soil fungal communities in the bulk soils of the topsoil layer were differentiated into five distinct clusters (Figure 4A). Furthermore, the unconstrained PCoA using Bray–Curtis distance revealed that the soil fungal communities in the topsoil of the examined forest types were clearly separated along the first axis (Table 1, Figure 4B). The first two PCoA axes accounted for 12.85% and 8.48% of the variation in soil fungal communities at the topsoil layer, respectively. The “Envfit” analysis demonstrated that the key driving factors influencing the soil fungal community in the topsoil layer were pH, SOC, AN, TN, SWC, C/P, and N/P.

The analysis of soil fungal communities at the topsoil layer across different forest types using NMDS ordination revealed distinct clustering into five separate groups (Figure 4A). This differentiation was further supported by adonis analysis (Table 1), which confirmed significant differences in fungal community structures at the topsoil layer among the forest types. Additionally, the PCoA indicated a clear shift in soil fungal community composition at the topsoil layer influenced by forest type (Figure 4B). The first two PCoA axes accounted for 12.85% and 8.48% of the variation in soil fungal communities, respectively. Envfit analysis identified several critical driving factors affecting soil fungal community composition at the topsoil layer, including soil pH, SOC, AN, TN, SWC, C/P, and N/P (Table 2).

#### 3.2.3. Indicator Species

The indicator species analysis identified several taxa as significant markers for specific forest types. *Russula crustose* and Boletales were indicators of the evergreen broad-leaved forests, while *Nectria* spp., *Nectria ramulariae*, *Clavulina castaneipes*, and *Sebacina dimitica* were indicators of coniferous forest. In the deciduous broad-leaved and evergreen broad-leaved mixed forests, the Archaeorhizomycetes, Archaeorhizomycetales, Archaeorhizomycetaceae, and Archaeorhizomyces served as key indicators. In the secondary forests, *Mortierella pseudozygospora*, *Mortierella sclerotiella*, *Anguillospora* spp., and *Auguillospora crassa* were indicators. In the plantation forest, the indicators included Trimorphomycetaceae, Saitozyma, *Saitozyma podzolica*, Tremellales, Hymenoscyphus, *Hymenoscyphus tetracladius*, Trichophaea, *Mortierella gamsii*, and O_GS11. These indicator species reflect the distinct ecological niches and environmental conditions characteristic of each forest type.

### 3.3. Co-Occurrence Networks

**Figure 5:**
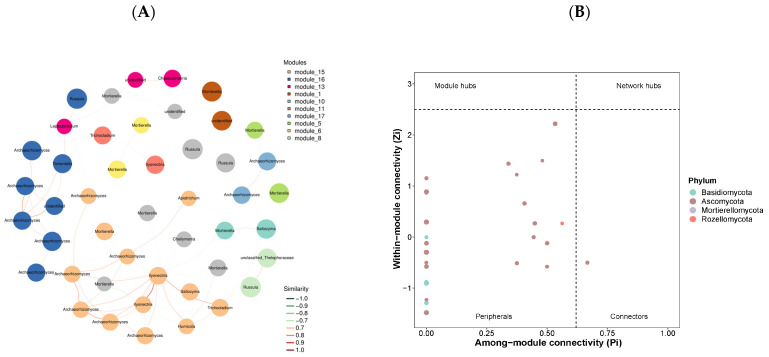

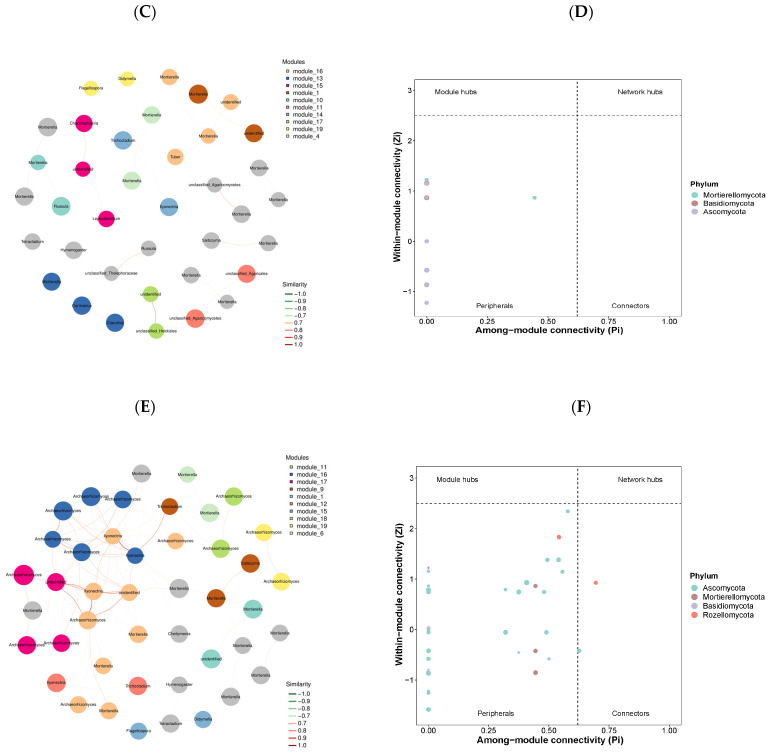

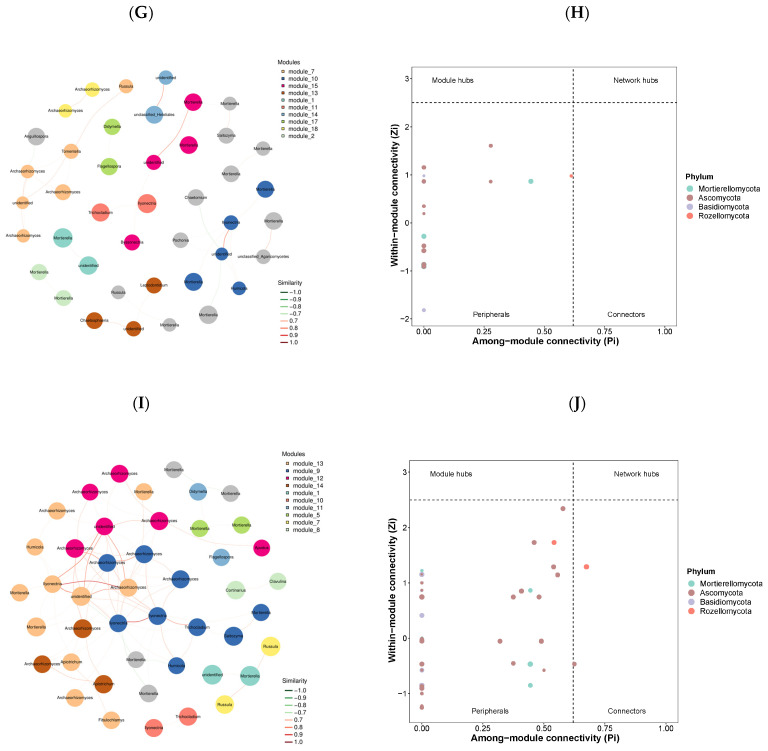
Co-occurrence network patterns and Zi-Pi plots of the top 50 soil fungal ASVs in evergreen broadleaved forest (**A**,**B**), coniferous forest (**C**,**D**), deciduous broadleaved and evergreen broadleaved mixed forest (**E**,**F**), secondary forests (**G**,**H**), and plantation forest (**I**,**J**). Nodes of the same color indicate that the species belong to the same phylum. Edges represent significant Spearman’s correlations (|r| ≥ 0.6 and *p* < 0.05), while red and green lines indicate positive and negative correlations, respectively.

Network analyses revealed that species co-occurrence exhibited distinct patterns across the five examined forest types (Figure 5A,C,E,G,I). The coniferous forest displayed a relatively simple network with 39 edges and 63 nodes. Network modularity was highest in the coniferous forest (0.942), followed by the secondary forest (0.841) and the evergreen broad-leaved forest (0.658), indicating varying degrees of compartmentalization in fungal interactions. Among the set of network topological features calculated, network complexity, as indicated by the average degree value, was considerably higher in the deciduous broad-leaved and evergreen broad-leaved mixed forest, plantation forest, and evergreen broad-leaved forest, compared to other forest types (Table 3). The keystone taxa were also significantly different across forest types (Figure 5B,D,F,H,J). 

### 3.4. Soil Fungal Community Assembly Processes

**Figure 6:**
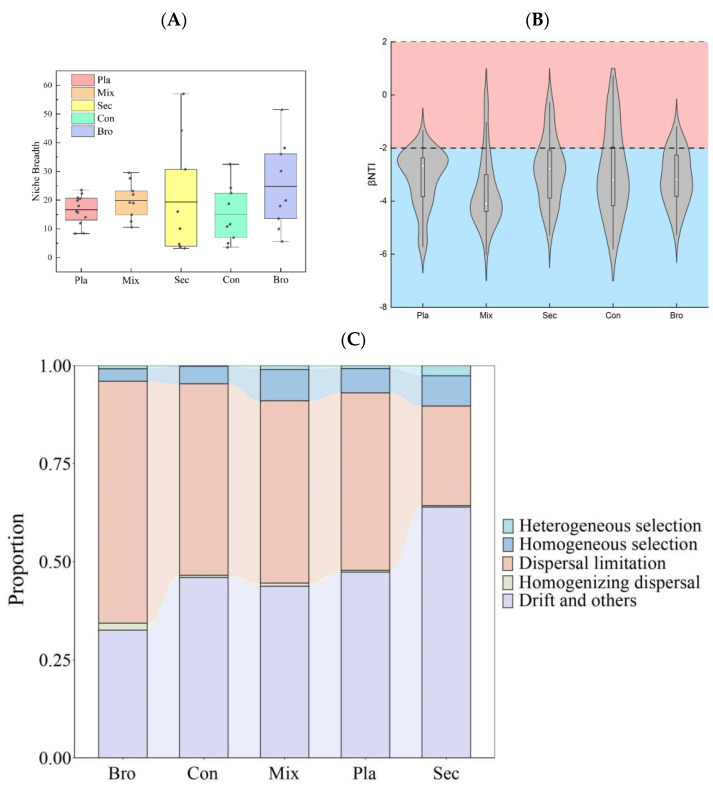

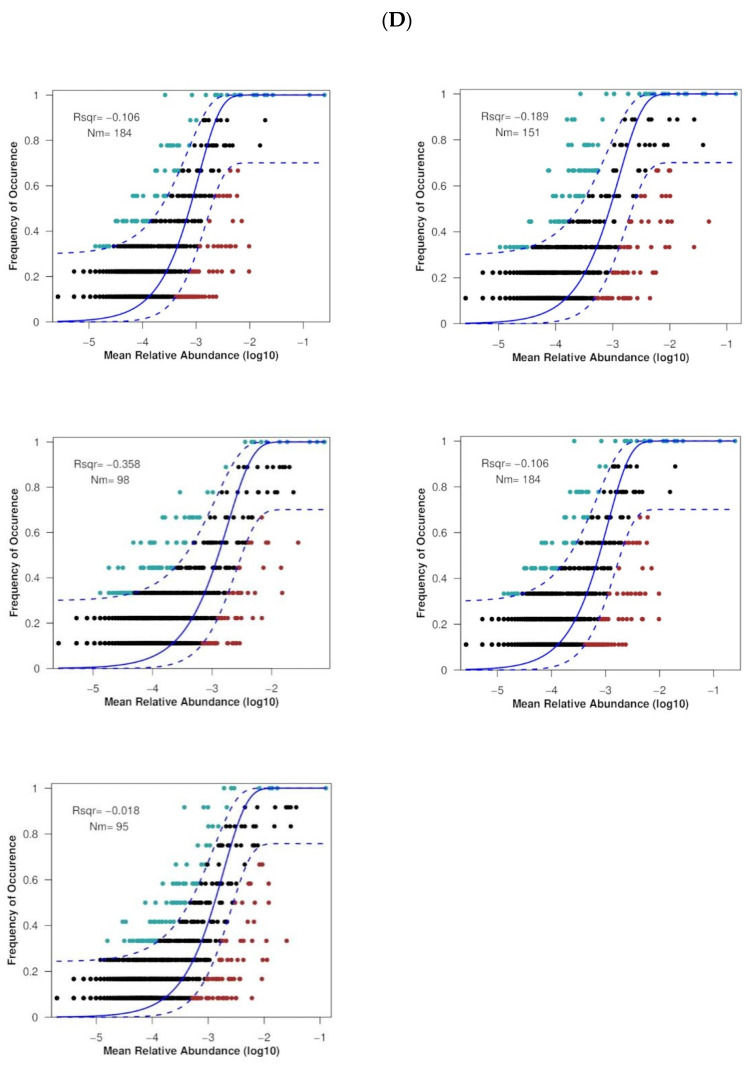
(**A**) Comparison of the ecological niche breadths of soil fungal communities in different forest types, (**B**) β-nearest taxon index (βNTI) of soil fungal communities in different forest types, (**C**) null model analysis of the community assembly processes. Deterministic processes, homogeneous + variable selection; stochastic processes, dispersal limitation + homogenizing dispersal + undominated processes; homogenizing, homogeneous selection + homogenizing dispersal; differentiating, variable selection + dispersal limitation. (**D**) Fit of Sloan’s neutral model for analysis of fungal community assembly. The solid blue lines indicate the best fit to the neutral model, and the dashed blue lines represent 95% confidence intervals around the model prediction. ASVs that occur more or less frequently than predicted by the neutral community model are shown in different colors. Rsqr indicates the fit to the neutral model, while the m value indicates community immigration rate. Bro, evergreen broadleaved forest; Con, coniferous forest; Mix, natural deciduous broadleaved and evergreen broadleaved mixed forest; Sec, secondary forest; Pla, plantation forest.

The niche width index of each soil fungal ASVs varied greatly among forest types, particularly for evergreen broad-leaved forest and secondary forest (Figure 6A). The betaNTI of soil fungal communities of the examined forest types were significantly below −2 (Figure 6B), indicating that soil fungal community assembly processes in this region were dominated by deterministic processes, exactly speaking homogeneous selection. In contrast, phylogenetic bin-based null model analysis revealed that the assembly processes were dominated by dispersal limitation, drift, and others (Figure 6C). Finally, the soil fungal community at the topsoil layer of all examined forest types did not fit well with the neutral model (Figure 6D).

## 4. Discussion

Soil fungi play a critical role in regulating plant diversity and productivity as well as delivering ecosystem services in forest ecosystem. The motivation of the present study is to comprehensively understand the linkages among soil, soil fungal community and forest stand quality, and to utilize soil microbiome information for rational forest management decisions ultimately.

### 4.1. Relative Abundances of Soil Fungi at Various Levels

The findings from the comprehensive analyses of ASV, phylum, and guild levels underscore the diverse and distinct soil fungal communities present in different forest types, reflecting the complex ecological interactions and environmental conditions within the Liziping Natural Reserve. In a previous study, the relative abundance of symbiotic fungi in the coniferous forest was found to be significantly lower than that in the broad-leaved forest [21]. However, the relative abundance of symbiotic fungi in the coniferous forest was comparable to that in the broad-leaved forest in the present study. The reasons for this discrepancy need further study. Basidiomycota and Ascomycota were positively correlated with the TC and TN contents [9], and ECM fungi was negatively correlated with soil TP and AP content [31]. By contrast, the relative abundances of Ascomycota and ECM fungi were positively correlated with soil pH and C/N in the present study.

### 4.2. Soil Fungal Community Diversity across Distinct Vegetation Types

One of the major findings of the present study is that no significant difference in α diversity, but a significant difference was observed in β diversity among soil fungal communities of different stand types. The results of the present study agree with previous studies that fungal community diversity in broadleaved forests is higher compared to coniferous forests [6,49,67]. Two likely reasons account for this pattern. On one hand, broadleaved forests contain more decomposable and lower concentrations of chemically complex compounds such as lignin than that of conifer forests [68,69]; on the other hand, broadleaved forests provide more favorable micro-environments for soil fungi [49]. Regarding α diversity, previous studies suggest that fungal community diversity across forest types. For instance, the Shannon index of soil fungal community was affected by vegetation type [48]. The discrepancy in the effects of forest type on α diversity of soil fungal communities across different studies can be attributed to several factors. Different studies are often conducted in regions with varying climates, elevations, and environmental conditions, which can significantly influence soil fungal communities. For example, tropical forests tend to have higher fungal diversity than temperate forests due to warmer and more stable climates [70]. Soil properties, including pH, nutrient content, moisture, and organic matter, can vary significantly between forest types and locations, affecting the fungal communities present [67,71]. Variations in sampling methods, DNA extraction techniques, sequencing platforms, and bioinformatics pipelines can lead to differences in the observed diversity of soil fungi. Studies using different primers or sequencing depths may capture different portions of the fungal community [1]. The proxy used to indicate α diversity was also distinct. For instance, a recent study proposed that the nonparametric richness estimators Chao1 and ACE should never be used with ASV data [72]. Fungal communities can be highly dynamic and subject to seasonal changes. Studies conducted at different times of the year may report different levels of α-diversity due to these temporal fluctuations [73]. The composition of tree species in a forest can influence the soil fungal community due to variations in litter quality, root exudates, and mycorrhizal associations [74].

The results of the present study are partly in agreement with previous studies which suggest that the diversity and composition of fungal communities are mostly associated with aboveground plant vegetation [9,75]. These findings based on the correlations between soil physicochemical properties and β diversity of soil fungal community emphasize the complex interplay between soil physicochemical properties and fungal community dynamics at the topsoil layer in different forest types. The observed variations in fungal diversity may be attributed to differences in soil properties, vegetation composition, and environmental conditions including differing microclimates among forest types [1]. Soil pH and TN were important for the fungal community structure in broad-leaved forests [76]. AN, TK, and pH mainly influenced the soil fungal community [77], and soil pH and electrical conductivity were the major driving factors of soil fungal community in subtropical forests [78]. Future studies should consider the effects of additional factors, such as stand age (not examined in the present study) on soil fungal community.

### 4.3. Co-Occurrence Network Patterns

Fungal species form complex intradomain interaction network, and the network complexity indicates fungal community stability [5,79,80]. In general, more connected networks are more efficient in soil functioning, such as carbon uptake and nutrient cycling [2,81]; complex co-occurrence networks had a stronger ability to resist environmental interference than simple networks [7,82,83]. In the present study, soil fungal communities in evergreen broad-leaved forests and deciduous broad-leaved and evergreen broad-leaved mixed forests showed high modularity and average path length, indicating their highly modular nature without distinct small-scale characteristics.

The networks exhibited characteristics of scale-free topology, with a few highly connected nodes (hubs) and many poorly connected nodes. This pattern suggests the presence of keystone taxa that play important roles in maintaining network stability and ecosystem functioning [84]. Additionally, the proportions of positive interaction edges of soil fungal networks were higher in evergreen broad-leaved forests and deciduous broad-leaved and evergreen broad-leaved mixed forests networks, indicating that fungal communities form tight organizations through cooperation, thus enhancing the complexity of community structure and ecosystem stability. This can be explained by a reduced competition between species, related to nutrient resources, and microorganisms may cooperate to adapt to similar niches [85].

### 4.4. Soil Fungal Community Assembly Processes

The findings of the present study indicated that both deterministic and stochastic factors contribute to fungal community assembly at the topsoil layer across different forest types, while dominated by dispersal limitation and ecological drift. Similar results are found elsewhere [86]. For instance, deterministic processes mainly influenced soil fungal community assembly in natural forests, whereas stochastic processes mainly influenced soil fungal community assembly in plantation forests [77,87]. The relative importance of these processes may also vary depending on the spatial scale, dispersal capacity of fungal taxa, and historical legacies [88].

Concordant with previous studies that reporting the frequency of microbial OTUs fit the neutral model poorly [40,57,89], the frequency of soil fungal ASVs did not fit the neutral model in the present study. These findings indicate that the stochastic processes represented by the neutral model are inadequate to explain the observed distribution patterns of soil fungal communities. Instead, deterministic factors such as environmental selection, biotic interactions, and historical contingencies likely play a more significant role in shaping these communities. This suggests that soil fungal diversity and distribution are more closely governed by specific ecological niches and the prevailing environmental conditions than by random dispersal and demographic stochasticity.

The frequency with which soil fungal ASVs deviate from neutral model predictions can be attributed to several ecological and biological factors. Neutral models in community ecology assume that all individuals of different species are ecologically equivalent, with community composition driven mainly by stochastic processes such as random birth, death, and dispersal events. However, several non-neutral processes often play significant roles in shaping soil fungal communities. Firstly, soil environments are highly heterogeneous, exhibiting variations in pH, moisture content, organic matter, nutrient availability, and other factors. Fungal ASVs exhibit varying ecological preferences and tolerances, leading to non-random distribution patterns that neutral models cannot account for. Secondly, fungi engage in various interactions, including competition, mutualism (e.g., mycorrhizal relationships with plants), and antagonism, which can significantly influence community structure [70]. These interactions can cause significant deviations from the predictions of neutral models. Thirdly, neutral models assume random and sufficient dispersal to homogenize communities, but dispersal can be limited by physical barriers or distances [90]. Fungal spores often do not disperse evenly across all potential habitats, resulting in spatially structured communities. Fourthly, specific environmental conditions may selectively favor certain fungal ASVs over others [1]. For instance, certain fungi may be better adapted to decomposing specific types of organic matter or thriving under moisture conditions. Such selective pressures result in non-neutral community composition patterns. Finally, fungal communities typically exhibit high functional diversity, with different species playing unique roles in ecosystem processes such as decomposition, nutrient cycling, and symbiotic relationships with plants [91].

### 4.5. Implications for Forest Ecosystem Conservation and Management

The findings underscore the significance of conserving diverse forest ecosystems to maintain soil fungal biodiversity and the associated ecosystem services. These results bear substantial implications for forest management and conservation strategies. Specifically, considering the diversity and composition of soil fungal communities across various forest types can inform management practices aimed at enhancing ecosystem resilience and productivity. Promoting tree species diversity in plantation forests, for instance, may enhance fungal diversity and soil health, thereby improving forest ecosystem services [92].

Furthermore, conserving keystone fungal taxa and their habitats is essential for maintaining network stability and ecosystem functioning within forests. Although the present study provides valuable insights into soil fungal communities across different forest types, it is not without limitations. The integration of molecular techniques with functional assays is recommended to advance the understanding of the ecological roles of different fungal taxa in forest ecosystems. Future research should aim to elucidate the mechanisms underlying the observed patterns of fungal diversity and assembly across forest types, including the roles of soil properties, microclimate, and multitrophic interactions among soil biota.

## 5. Conclusions

In the topsoil layer of typical forests within the Liziping Natural Reserve, soil fungal communities were predominantly composed of Ascomycota, Basidiomycota, and Mortierellomycota. While no significant differences in α diversity were detected, there were notable differences in β diversity among the fungal communities in various forest stand types. Key factors influencing these soil fungal communities included soil pH, SOC, TN, TP, and N/P. Each forest type exhibited a distinct intradomain soil fungal co-occurrence network. The structure of the fungal community in the topsoil layer was primarily driven by stochastic processes, especially dispersal limitation.

## Figures and Tables

**Table 1:** The significant difference in β diversity of soil fungal community across forest types as examined by permutational analyses of variance (PERMANOVA) using *adonis2* in the vegan package in R.

Object	*Adonis2*		
	*F*	*R* ^2^	*Adjusted-P*
Among Groups	2.8721	0.2231	0.001
Mix vs. Sec	2.5124	0.1357	0.001
Mix vs. Con	2.0193	0.1121	0.025
Mix vs. Bro	1.9657	0.1094	0.044
Sec vs. Con	2.0201	0.1121	0.008
Sec vs. Bro	3.0971	0.1622	0.001
Con vs. Bro	2.2155	0.1216	0.001
Pla vs. Mix	3.1503	0.1645	0.001
Pla vs. Sec	4.6003	0.2233	0.001
Pla vs. Con	4.5123	0.2200	0.001
Pla vs. Bro	3.2643	0.1694	0.001

**Note:** Bro, evergreen broadleaved forest; Con, coniferous forest; Mix, natural deciduous broadleaved and evergreen broadleaved mixed forest; Sec, secondary forest; Pla, plantation forest. All *p*-values were adjusted with the “FDR” method.

**Table 2:** Correlations between soil fungal community similarity and individual soil physicochemical property based on the Envfit of the principal co-ordinates analysis (PCoA).

Variable	PCoA1	PCoA2	R^2^
pH	−0.89511	−0.44584	0.2815
AP	0.99812	0.06127	0.0205
SOC	−0.47273	0.88121	0.1621
TP	0.48448	−0.8748	0.0202
TN	0.01229	0.99992	0.2495
AN	−0.00816	0.99997	0.1985
SWC	−0.22794	0.97368	0.2782
C/N	−0.81507	−0.57937	0.1093
C/P	−0.36499	0.93101	0.2254
N/P	−0.02049	0.99979	0.3836

**Note:** AN, available nitrogen; AP, available phosphorus; C/N, the ratio of soil organic carbon to total nitrogen; C/P, the ratio of soil organic carbon to total phosphorus; N/P, the ratio of total nitrogen to total phosphorus; SOC, soil organic carbon; SWC, soil water content; TN, total nitrogen; TP, total phosphorus.

**Table 3:** Topological coefficients of soil fungal co-occurrence networks under typical forest types in the Liziping Reserve.

Type	Objective	Bro	Con	Mix	Sec	Pla
Empirical network	Nodes	69	63	87	60	89
	Edges	87	39	121	52	121
	Network diameter	4.662	2.675	5.994	2.676	5.99
	Modularity	0.658	0.942	0.583	0.841	0.576
	Graph density	0.037	0.020	0.032	0.029	0.031
	ANND	4.225	1.408	4.883	2.477	4.785
	Average path length	2.683	1.458	3.023	1.845	3.07
	Between centrality	10,950	477	28,708	1541	31,012
	Closeness centrality	0.388	0.055	0.458	0.141	0.474
	Degree centralization	861	111	1150	316	1182
Random network	Network diameter	9.10 ± 0.88	8.60 ± 2.01	9.40 ± 1.35	12.30 ± 2.11	9.50 ± 1.08
	Modularity	0.575 ± 0.014	0.795 ± 0.039	0.557 ± 0.022	0.666 ± 0.030	0.580 ± 0.020
	ANND	3.451 ± 0.203	2.145 ± 0.170	3.771 ± 0.158	2.737 ± 0.210	3.608 ± 0.121
	Average path length	4.160 ± 0.214	3.528 ± 0.688	4.136 ± 0.193	5.197± 0.688	4.292 ± 0.231
	Between centrality	27,114.22 ± 8738.24	6092.80 ± 2835.92	36,778.26 ± 11,019.72	23,167.14 ± 6406.49	52,255.11 ± 1152.88
	Closeness centrality	1.349 ± 0.394	0.251 ± 0.105	1.436 ± 0.371	0.723 ± 0.092	1.559 ± 0.421
	Degree centralization	288.300 ± 80.006	192.900 ± 30.431	367.000 ± 100.460	232.00 ± 50.596	389.900 ± 97.945

**Note:** ANND, average nearest neighbor degree;; Bro, evergreen broadleaved forest; Con, coniferous forest; Mix, natural deciduous broadleaved and evergreen broadleaved mixed forest; Sec, secondary forest; Pla, plantation forest.

## Data Availability

The raw sequence data from this study were deposited in the SRA at the NCBI database.

## Supplementary Materials

The following supporting information can be downloaded at: https://www.mdpi.com/article/10.3390/microorganisms12091915/s1. Figure S1: Spearman correlations between relative abundance of soil fungal phylum, guilds, trophic modes and soil physicochemical properties. The values in the circle are *r*-values of spearman correlation and the “*” “**” “***” represent the significances. *, *p* < 0.05; **, *p* < 0.01; ***, *p* < 0.001; Figure S2: ASV numbers of pathotroph fungi (A), saprotroph fungi (B), symbiotroph fungal (C) and unassigned fungi (D). The horizontal bars within boxes represent medians. The tops and bottoms of boxes represent the 75th percentiles and 25th percentiles, respectively; Figure S3: Linear discriminant analysis effect size (LEfSe) analyses of fungal communities in soil of typical forest types (LDA significance threshold >3.0); Figure S4: Co-occurrence network patterns of fungal communities at the genera level in deciduous broadleaved forest (A), coniferous forest (B), deciduous broadleaved and evergreen broadleaved mixed forest (C), secondary forests (D) and plantation forest (E). Only nodes (genus) that were significantly correlated with each other (|ρ| > 0.60; *p* < 0.05) were connected (edges). The sizes of the nodes are proportional to node degree.
