# Supplementary material for: Soil Fungal Community Diversity, Co-Occurrence Networks, and Assembly Processes under Diverse Forest Ecosystems
Source: Microorganisms. 2024 Sep 20;12(9):1915. doi: 10.3390/microorganisms12091915 (PMC11433935; doi:10.3390/microorganisms12091915)

microorganisms

Supplementary Materials

Figure S1. Spearman correlations between relative abundance of soil fungal phylum, guilds, trophic modes, and soil physicochemical properties. The values in the circle are R-values of the Spearman correlations and the "*", "**", and "***" represent the significances. *, p<0.05; **, p<0.01; ***, p<0.001.

(A) **(B)**

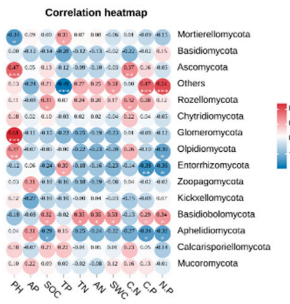

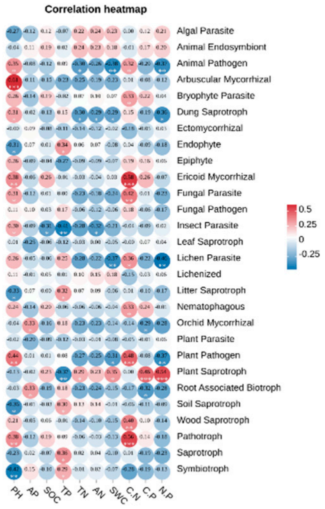

Figure S2. ASV numbers of pathotroph fungi (A), saprotroph fungi (B), symbiotroph fungal (C), and unassigned fungi (D). The horizontal bars within boxes represent medians. The tops and bottoms of boxes represent the 75th percentiles and 25th percentiles, respectively.

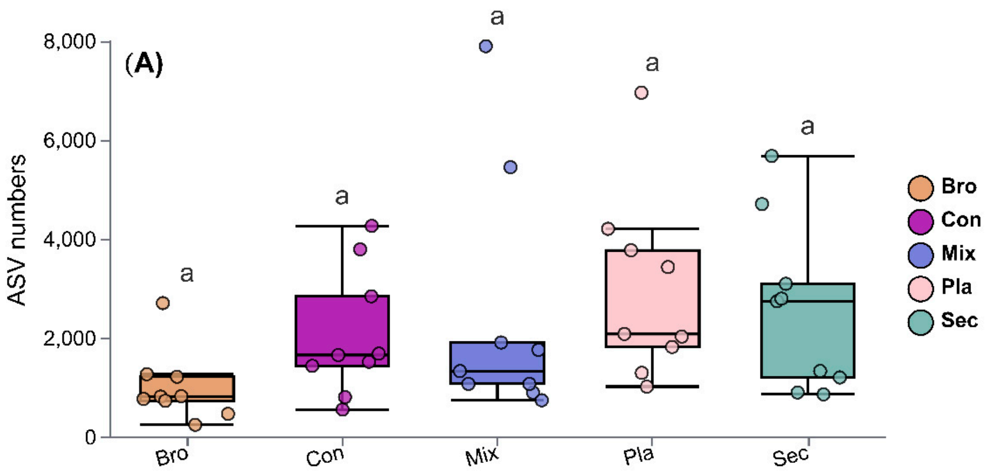

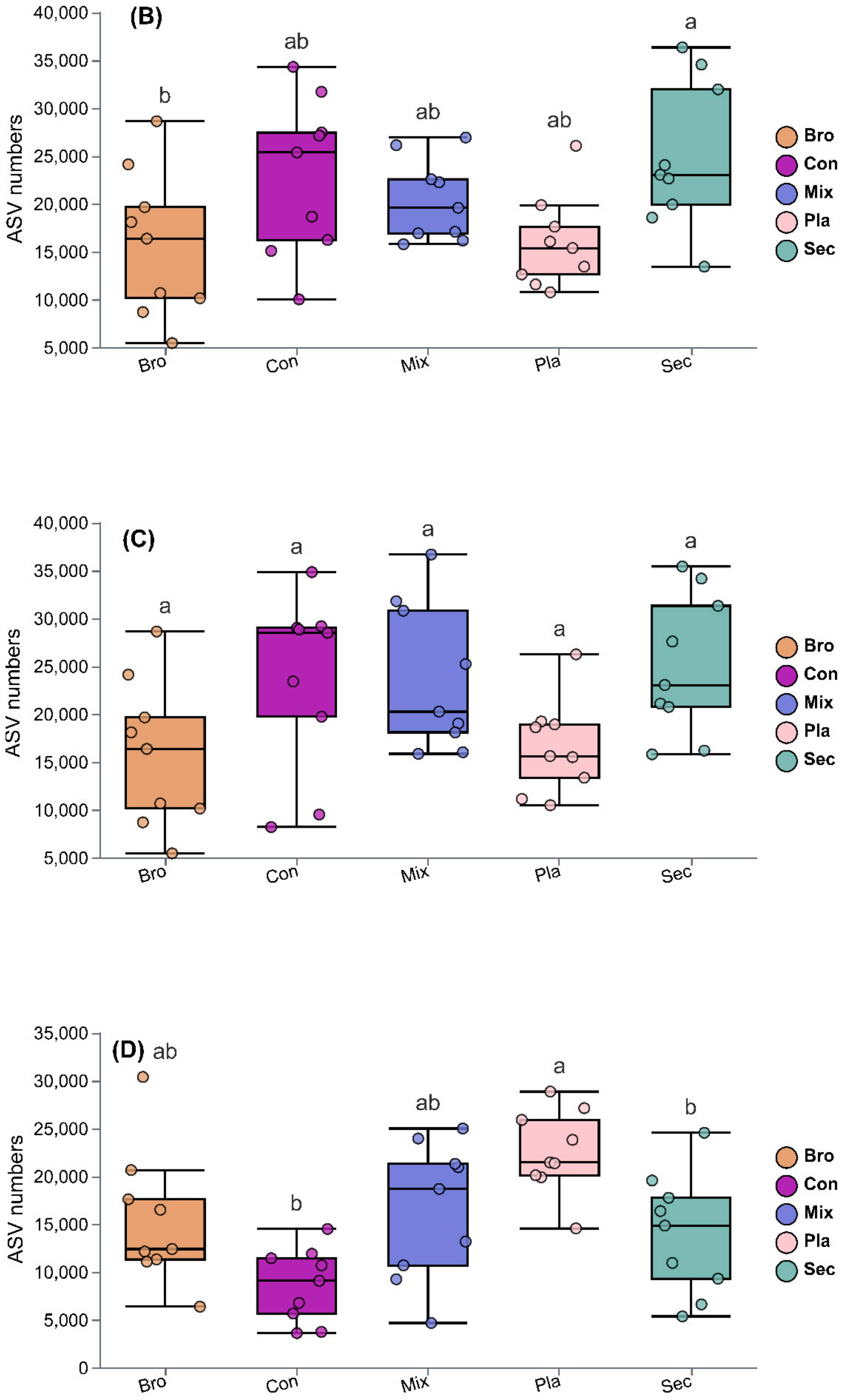
(B)
ASV numbers
40,000
35,000
30,000
25,000
20,000
15,000
10,000
5,000
b
ab
ab
ab
a
Bro
Con
Mix
Pla
Sec
(C)
a
a
a
a
a
(D)
ab
b
ab
a
b
0

Figure S3. Linear discriminant analysis effect size (LEfSe) analyses of fungal communities in soil of typical forest types (LDA significance threshold >3.0).

(A)

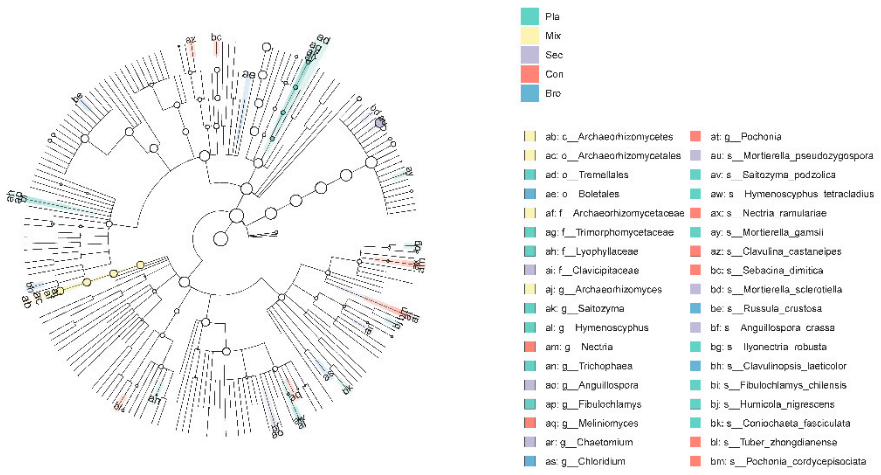

(B)

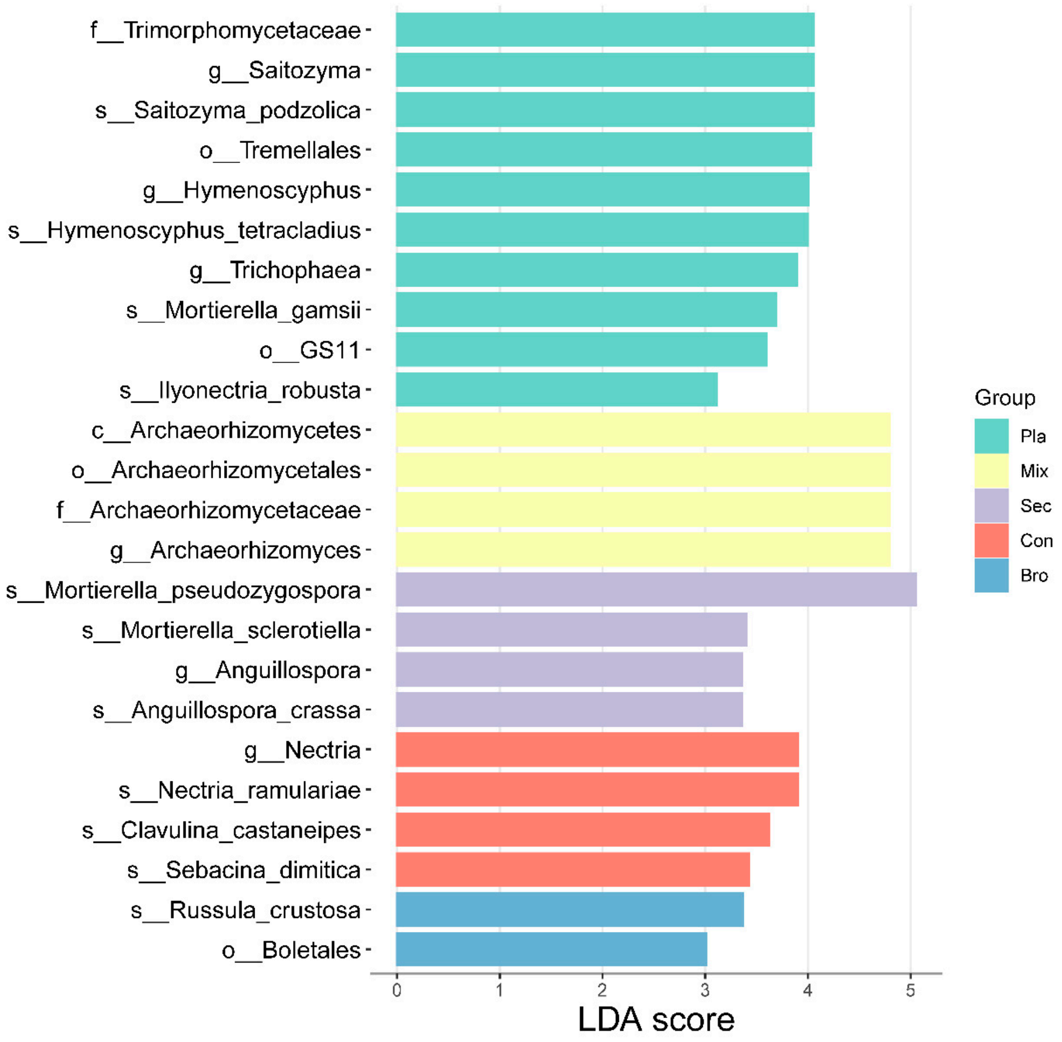

Figure S4. Co-occurrence network patterns of fungal communities at the genera level in deciduous broadleaved forest (A), coniferous forest (B), deciduous broadleaved and evergreen broadleaved mixed forest (C), secondary forests (D), and plantation forest (E). Only nodes (genus) that were significantly correlated with each other (|ρ| > 0.60; P < 0.05) were connected (edges). The sizes of the nodes are proportional to node degree.

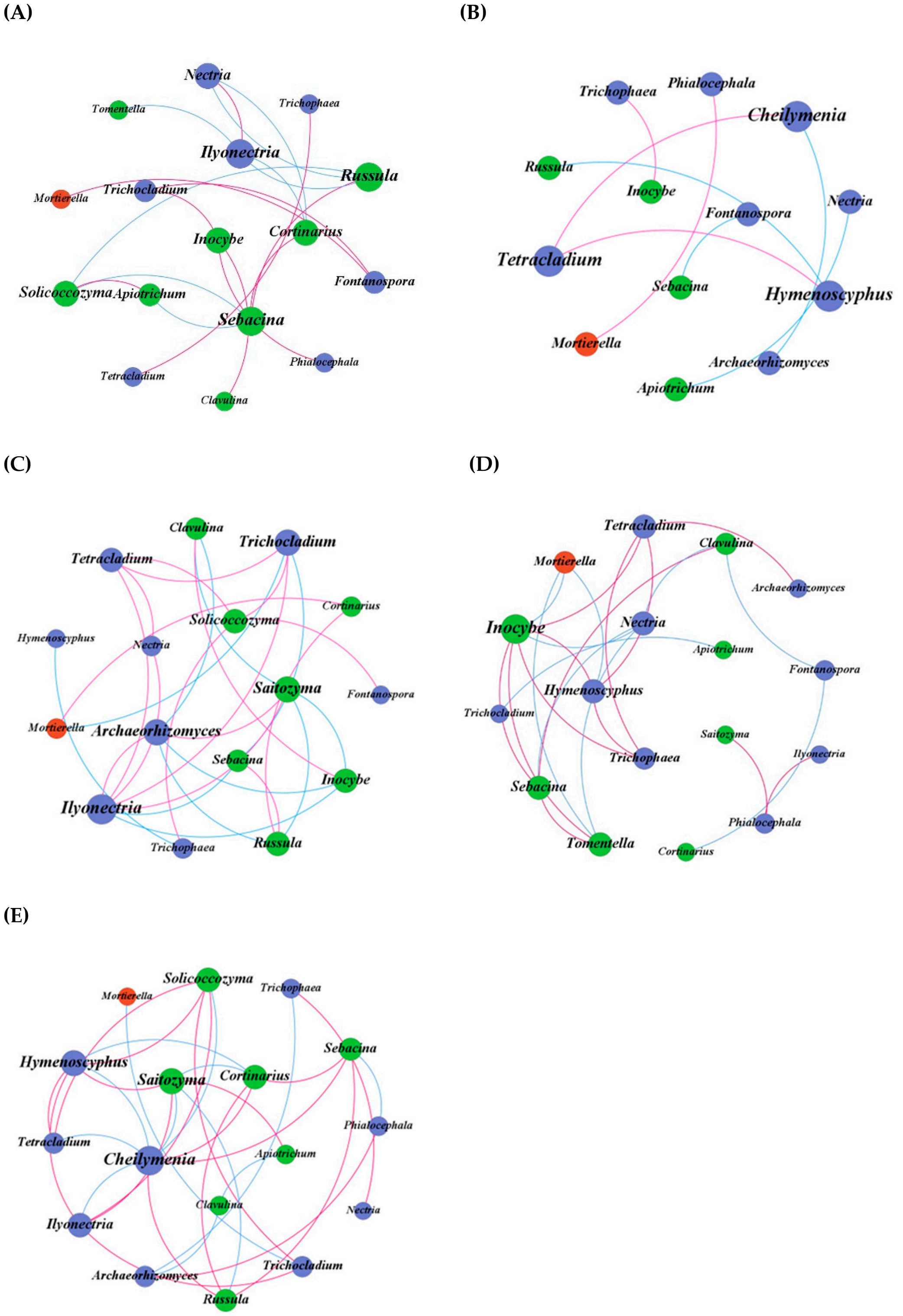

Supplement: Supplementary file 1 [file microorganisms-12-01915-s001.zip › microorganisms-3185195-supplementary.pdf]
